# Anesthetic Management of Cesarean Delivery in a Patient With Ellis–van Creveld Syndrome

**DOI:** 10.14740/jmc5288

**Published:** 2026-07-28

**Authors:** Devaki Kalvapudi, Syed Ammar Shah, Hardeep Singh

**Affiliations:** a Internal Medicine, Northeast Georgia Medical Center, Gainesville, GA 30501, USA; b Northeast Georgia Medical Center, Gainesville, GA 30501, USA; c GME Research, Northeast Georgia Medical Center, Gainesville, GA 30501, USA

**Keywords:** Ellis–van Creveld syndrome, Cesarean section, Neuraxial anesthesia, Skeletal dysplasia, High-risk pregnancy

## Abstract

Ellis–van Creveld (EVC) syndrome is a rare autosomal recessive skeletal dysplasia characterized by disproportionate short stature, skeletal abnormalities, craniofacial and dental anomalies, and a high prevalence of congenital cardiovascular disease. Pregnancy in patients with EVC syndrome presents unique anesthetic challenges due to altered airway anatomy, abnormal spinal anatomy affecting neuraxial techniques, restrictive pulmonary physiology, potential cardiac dysfunction, and resultant hemodynamic complications. Evidence guiding anesthetic management for this population remains limited. We present the case of a 23-year-old pregnant patient with a heterozygous *EVC2* gene variant, who presented for repeat cesarean section. After comprehensive pre-anesthetic evaluation, neuraxial anesthesia with spinal blockade was selected to avoid airway manipulation and minimize hemodynamic stress. Adequate surgical anesthesia was achieved with hyperbaric bupivacaine, and the patient remained hemodynamically stable throughout the intraoperative and postoperative periods without complications. This case highlights the importance of individualized anesthetic planning in patients with rare skeletal dysplasia undergoing cesarean section. Careful assessment of airway, spine, and cardiac function allows safe use of neuraxial anesthesia and may reduce perioperative morbidity to ensure best operative outcomes.

## Introduction

Ellis–van Creveld (EVC) syndrome is a rare autosomal recessive skeletal dysplasia caused by pathogenic variants in the *EVC* or *EVC2* genes, which play a role in hedgehog signaling and endochondral bone development [1, 2]. The syndrome is characterized by disproportionate short stature, postaxial polydactyly, short ribs, craniofacial and dental abnormalities, and a high incidence of congenital heart disease [1, 2]. The estimated prevalence is fewer than 1 in 100,000 live births, though higher rates have been reported in certain genetically isolated populations [1].

Cardiovascular involvement is a major determinant of morbidity and mortality in EVC syndrome. Congenital defects such as atrioventricular septal defects, atrial septal defects, and single atrium are commonly reported, and affected individuals may also develop acquired cardiac disease later in life [3]. Skeletal abnormalities, including thoracic restriction, may contribute to restrictive lung disease. Short stature, narrowed intervertebral spaces or scoliosis can make neuraxial anesthesia unpredictable, further complicating anesthetic management. Craniofacial and dental abnormalities may also increase the risk of difficult mask ventilation or tracheal intubation [3].

Pregnancy introduces additional physiological challenges, including increased oxygen consumption, decreased functional residual capacity, airway edema, and significant hemodynamic changes [4]. In obstetric anesthesia, neuraxial techniques are generally preferred for cesarean delivery; however, abnormal spinal anatomy and unpredictable spread of local anesthetic may complicate their use in patients with skeletal dysplasia [5].

Currently, limited research exists regarding anesthetic management of pregnant patients with EVC syndrome. Most evidence is derived from isolated case reports. This case contributes additional evidence supporting the feasibility of neuraxial anesthesia for cesarean delivery in patients with EVC syndrome when comprehensive pre-anesthetic assessment and multidisciplinary planning are employed.

## Case Report

A 23-year-old gravida 3, para 1 woman at 39 weeks gestation presented for scheduled repeat cesarean section. She has a known diagnosis of EVC syndrome with disproportionate short stature and characteristic achondroplasia like features and dental abnormalities. She was born with extra digits on her hands and feet, and previously underwent polydactyly surgical repair in her childhood. She also had poor dentition, with a history of full mouth teeth extraction and permanent denture placement.

Prior genetic testing revealed a heterozygous variant in trans in the *EVC2* gene when she was diagnosed with EVC syndrome. She has previously undergone testing with skeletal dysplasia panel, which revealed two heterozygous pathogenic variants in trans in the *EVC2* gene at c.619G>T and c.893del, consistent with clinical diagnosis of EVC syndrome. She was also found to have an abnormality in the *OBSLI* gene of uncertain significance, which is associated with the 3M syndrome, characterized by short stature, and craniofacial and skeletal anomalies [6]. A variant of unknown significance in the *SRCAP* gene at c.5518C>T was also identified in this patient. This gene is classically associated with the autosomal dominant Floating-Harbor syndrome or other neurodevelopmental disorders; however, the absence of characteristic phenotypic features in this patient suggested no clinical relevance [7].

**Figure 1:**
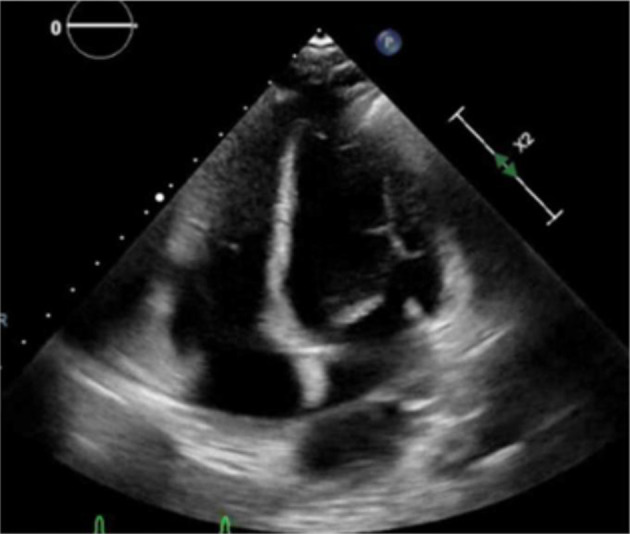
Complete transthoracic echocardiography showing ejection fraction of 55–60% with no significant valvular abnormalities.

The patient had regular antenatal testing, with targeted fetal anatomical surveys. Fetal echocardiogram revealed no fetal abnormalities, with appropriate growth. Prior obstetric history was significant for an uneventful cesarean delivery via neuraxial anesthesia 2 years prior. The patient reported prior cardiac history, stating that she had two episodes of myocardial infarctions, for which she was treated at an outside facility. Upon evaluation by cardiology, she denied that she underwent revascularization procedures or surgery. Electrocardiogram did not show any ST segment changes, q waves, or other anomalies, and she had no complaints of chest pain or shortness of breath. A follow-up transthoracic echocardiography demonstrated preserved left ventricular systolic function with an ejection fraction of 55–60% and no significant valvular abnormalities or congenital defects (Fig. 1). She was started on ASA 81 mg daily, but later discontinued. There were plans to obtain cardiac computed tomography (CT); however, the patient was subsequently lost to cardiac follow-up and the etiology of her chest pain ultimately was undiagnosed.

On pre-operative evaluation, she had short stature (height 137 cm, weight 55 kg, body mass index 29.6 kg/m^2^), short limbs in relation to the trunk, with no gross chest cavity abnormalities. Pre-anesthetic airway assessment included Mallampati score 3, full mouth opening, intact dentition, and full neck range of motion. No obvious cervical or lumbar spine abnormalities were noted, and no formal pulmonary assessment was made as she did not appear to have a narrow or short thoracic cavity. Baseline vital signs showed heart rate of 78/min, blood pressure (BP) 112/68 mm Hg, and room air saturation of 99%. After discussion with OBGYN, cardiology, and anesthesia teams, neuraxial anesthesia was selected as the primary plan to avoid airway manipulation and reduce hemodynamic stress. Airway manipulation could have been complicated by damage to previously placed permanent dentures. Neuraxial access carried a potential for technical difficulty due to short stature, possible kyphoscoliosis, and narrowed interlaminar spaces. Ultrasound-guided assessment of the lumbar spine was considered as an adjunct to surface landmark palpation to assist with interspace identification. Although not a significant physical finding in this patient, potential restrictive thoracic physiology also played a consideration in choice of neuraxial techniques over general anesthesia. As a backup, a general anesthesia plan for rapid sequence intubation (RSI) intubation with ketamine and succinyl choline was planned, with immediate access to a difficult airway cart containing instruments for video laryngoscopy and fiberoptic intubation nearby. Vasopressor support with phenylephrine was kept ready if high spinal block and resultant hypotension were to happen.

Spinal anesthesia was performed successfully, achieving a sensory block to the T4 dermatome using 1 mL of 0.75% hyperbaric bupivacaine. Bupivacaine was selected due to its established safety profile in obstetric anesthesia and predictable sensory block characteristics when administered intrathecally [8]. The dose was intentionally reduced below standard obstetric dosing, guided by the patient’s short stature and the anticipated reduction in lumbosacral cerebrospinal fluid (CSF) volume. Intravenous acetaminophen 1 g, 25 µg fentanyl, and 15 mg ketorolac was administered as an adjunct for multimodal analgesia.

The patient remained hemodynamically stable throughout the procedure without need for vasopressor support. The cesarean section and postoperative recovery were uneventful, and no anesthetic complications were observed in the post-anesthesia care unit.

## Discussion

This case demonstrates successful neuraxial anesthetic management of cesarean delivery in a patient with EVC syndrome and significant cardiac history. Patients with skeletal dysplasia present unique anesthetic challenges due to anatomical and physiological variations that affect airway management, neuraxial techniques, respiratory mechanics, and cardiovascular reserve [5].

Airway evaluation is critical in EVC syndrome, as craniofacial abnormalities, dental anomalies, and limited neck mobility may increase the risk of difficult mask ventilation or intubation [5]. Although this patient had acceptable mouth opening and neck mobility, a Mallampati class III airway warranted careful planning and preparation for difficult airway management. Avoidance of airway manipulation through neuraxial anesthesia was therefore advantageous.

Neuraxial anesthesia in patients with skeletal dysplasia may be complicated by abnormal vertebral anatomy, technical difficulty, and unpredictable spread of intrathecal local anesthetics [5]. Despite these concerns, spinal anesthesia provided reliable surgical anesthesia in this case without excessive cephalad spread or hemodynamic instability. Careful dosing was selected in consideration of the patient’s short stature and reduced cerebrospinal fluid volume, which can increase cephalad spread of local anesthetic [8].

Cardiac disease is a prominent feature of EVC syndrome and significantly influences anesthetic risk [3]. Although our patient’s cardiac history was unknown, pre-anesthetic evaluation did not show any significant murmurs or anatomical abnormalities. Congenital heart disease has been reported in up to 60% of affected EVC individuals, with atrial septal defects and atrioventricular canal defects being the most common [3]. However, our patient’s reported history of myocardial infarction in the absence of structural abnormalities is unique. Myocardial infarction in a 23-year-old without traditional cardiovascular risk factors is unlikely to represent atherosclerotic disease. The *EVC2* gene encodes a core component of the ciliary hedgehog signaling complex, which is essential for embryonic coronary vascular development and angiogenesis [9]. Its disruption may predispose to subclinical coronary microvascular anomalies not detectable on echocardiography, which could explain the possible etiology of our patient’s cardiac events [9]. Definitive characterization would require coronary CT angiography, which could not be completed prior to her current presentation.

Pregnancy-related increases in blood volume and cardiac output may exacerbate underlying cardiac pathology [4]. In this patient, preserved left ventricular systolic function supported the safe use of spinal anesthesia, as patients with normal ejection fraction are better able to tolerate the reductions in systemic vascular resistance associated with neuraxial blockade [10].

Published literature on anesthetic management in EVC syndrome remains limited to isolated case reports and few preliminary guidelines. Management strategies upon review of some reports were chosen with relation directly to the patient’s age and specific phenotypic burden. A significant portion of the EVC literature focuses on early childhood presentations, including corrective surgeries for post-axial polydactyly, dental extractions for neonatal teeth or delayed eruptions, and lower-limb orthopedic osteotomies to correct progressive genu valgum [11]. Pediatric case reports also focused on cardiac anomaly repairs. In these pediatric cohorts, general anesthesia is often preferred due to patient age and complexity of medical conditions [12–14]. Conversely, adult presentations often demand complex cardiac anatomical repairs, such as the correction of a single atrium or atrioventricular canal defects, where general endotracheal anesthesia with advanced monitoring techniques was used [10]. In obstetric management, the literature reflects a delicate balance between general and neuraxial techniques. While neuraxial anesthesia is typically preferred for cesarean deliveries to avoid difficult airway manipulation, severe baseline cardiac anomalies have frequently forced clinicians to opt for general anesthesia instead. In such obstetric cases, general anesthesia is chosen to precisely control systemic vascular resistance and avoid the profound, rapid hypotension associated with standard single-shot spinal anesthesia, which could catastrophically worsen right-to-left shunts or compromise myocardial perfusion [15]. However, choosing a neuraxial approach does not guarantee an uncomplicated course; several case reports highlight significant technical challenges with spinal anesthesia in EVC patients. Due to the unpredictable nature of skeletal dysplasia, narrow interspinous spaces, and implicit vertebral anomalies, clinicians have documented a high incidence of patchy, asymmetric, or incomplete anesthetic blocks and failed epidural catheter insertions [16]. This erratic spread of local anesthetic often requires emergent conversion to general anesthesia or supplementary local infiltration, reinforcing the necessity of a pre-planned, airway contingency plan [17].

This case adds to existing evidence supporting the feasibility of neuraxial anesthesia for cesarean delivery in carefully selected patients when comprehensive pre-anesthetic assessment and contingency planning are employed. This report describes a single case and may not be generalizable to all patients with EVC syndrome, particularly those with severe congenital cardiac defects or significant thoracic restriction [2]. Additionally, the patient carried a heterozygous variant rather than classic homozygous mutations, which influenced phenotypic severity. Although a *SRCAP* variant was identified, its known association with Floating-Harbor syndrome and absence of corresponding clinical features in this patient suggest that it did not contribute meaningfully to the anesthetic or obstetric presentation [7]. Further reports and case series with tailored approach to anesthetic management for each patient are needed to better define optimal anesthetic strategies in this rare population.

### Conclusion

This case highlights the importance of individualized anesthetic planning for pregnant patients with EVC syndrome undergoing cesarean section. Comprehensive assessment of airway anatomy, spinal anatomy, and cardiac function allowed safe and effective use of neuraxial anesthesia while minimizing perioperative risk for a patient with rare skeletal dysplasia. Early multidisciplinary collaboration and careful preoperative risk calculation are key to successful anesthetic management in this high-risk population. This case also adds to the limited body of literature that neuraxial anesthesia can be safely utilized in patients with skeletal dysplasias.

## Data Availability

The data supporting the findings of this study are available from the corresponding author upon reasonable request.
